# Psychosocial Problems and Condomless Anal Sex among Transgender Women in Two Cities of China: Study Based on the Syndemic Framework

**DOI:** 10.3390/ijerph192316161

**Published:** 2022-12-02

**Authors:** Danni Xia, Yingjie Chen, Ruijie Chang, Chen Xu, Xiaoyue Yu, Yujie Liu, Hui Chen, Rongxi Wang, Shangbin Liu, Xin Ge, Yuxuan Wang, Ajuan Liang, Fan Hu, Yong Cai, Ying Wang

**Affiliations:** 1 School of Public Health, School of Medicine, Shanghai Jiao Tong University, No. 227, South Chongqing Road, Shanghai 200025, China; 2 Reproductive Medical Center, Tongji Hospital, Tongji University School of Medicine, Shanghai 200065, China

**Keywords:** transgender women, psychosocial problems, condomless anal sex, syndemic

## Abstract

Studies examining the association between psychosocial problems and condomless anal sex (CAS) among transgender women (TGW) are rare. In this study, close attention was paid to the effect of co-occurring psychosocial problems on sexual risk behaviors. A cross-sectional study, including 247 TGW, was conducted in Kunming and Shenyang, China. The prevalence of condomless anal sex among TGW in the previous 6 months was 30.8%. Most of the psychosocial factors were associated with one another in bivariate logistic regression models. Low self-esteem (ORm = 2.99, 95% CI = 1.25–7.18), sexual compulsivity (ORm = 2.13, 95% CI = 1.13–4.00), and intimate partner violence (ORm = 2.21, 95% CI = 1.19–4.11) were discovered to be related to condomless anal sex in the multivariate regression model. No significant interactive effects of the syndemic factors on condomless anal sex were detected. More programmatic and effective HIV prevention interventions targeting psychosocial problems are required to reduce HIV infection within the population.

## 1. Introduction

Transgender women (TGW) are individuals whose gender identities or expressions are inconsistent with the male gender they were assigned at birth. Globally, TGW may experience the highest rates of human immunodeficiency virus (HIV) infection in any population and bear the disproportionate burden of disease [1]. A systematic review reported that the worldwide prevalence of HIV among TGW is 19.1%, with the odds ratio of infection being 48.8 times that among other adults of reproductive age [2]. In China, data on HIV prevalence among TGW are limited. One reason is that TGW are often coded as men who have sex with men (MSM) in routine HIV surveillance [3], and another is their poor HIV test uptake due to social discrimination, stigma, lack of social support, accessibility of health care (include HIV testing), and fear of disclosure [4]. A few studies have suggested that the high HIV prevalence among TGW is a concern. In 2018, Yan et al. [1] carried out a cross-sectional study recruiting 250 TGW in two cities of eastern China, among which 14.8% were detected to be HIV positive. Another study among 198 TGW sex workers indicated that the HIV prevalence was 27.8% [5].

Consistent condom use is one of the recommendations for preventing the spread of HIV [6]. Compared with people who did not use condoms consistently or at all, those who consistently used condoms had a 10 to 20 times lower odds of contracting the virus when exposed to it [7]. The effectiveness of condoms is confirmed both in heterosexual and in homosexual populations [8,9]. However, studies have shown that the prevalence of condomless anal sex (CAS) among TGW is much higher than in other sexual/gender minorities and the general population [10,11,12]. In India, a survey of TGW and MSM informed that inconsistent condom use with any type of male partners in the past month among TGW was 50.7%, which is higher than 44.4% reported by MSM [13]. 

As a minority population, TGW are often the victims of discrimination, exclusion, stigma, and violence from society. Transgender people are particularly vulnerable to mental health problems and psychological distress [14], and there is a high prevalence of depression, anxiety, low self-esteem, and loneliness among this population [13,14,15,16,17]. Within the literature, the rates of depression in transgender individuals ranged from 48% to 62% [14]. In the United States, the lifetime prevalence of depression was estimated to be as high as 62% compared to 16.6% in the general population [18]. According to a systematic review of the transgender population, the prevalence of anxiety disorder varied from 17% to 68% [19]. Bouman et al. [20] found that transgender individuals have a nearly three-fold increased risk of possible anxiety disorders than the general population. Numerous studies have noted that transgender communities are at increased risk of violence and injury, including physical and sexual abuse [15,21]. Data from a systematic review showed that the median lifetime prevalence of physical intimate partner violence (IPV) was 37.5%, the lifetime sexual IPV was 25.0% among transgender individuals, and they were 1.7 times more likely to experience any IPV compared to cisgender individuals [22]. According to previous studies, psychosocial problems are linked to the sexual risk behaviors of TGW, such as CAS, thus increasing the risk of HIV acquisition [23,24,25,26,27]. In Lima, Peru, a survey of 328 TGW found an association between physical IPV and condomless receptive anal sex (CRAS) [28]. Those who suffer from IPV find it often difficult to negotiate condom use with their partners due to fear of violence and inequalities [29]. Other psychosocial problems, such as low self-esteem [30], depression [31], loneliness [26], and sexual compulsivity [32], were also found to be associated with CAS.

A single psychosocial problem is inadequate to account for the high level of sexual risk behavior, and it is imperative to pay close attention to the effect of co-occurring psychosocial problems on behavior. In recent years, syndemic theory has gained much attention as an approach to understanding and addressing key issues in public health [33]. The concept of syndemic was first proposed to explain a series of intertwined and mutually reinforcing epidemics that result in a disproportionate burden of disease in a population [34]. Singer defined “syndemic” as “population-level clustering of social and health problems,” which meet three criteria: “(1) two (or more) diseases or health conditions cluster within a specific population; (2) contextual and social factors create the conditions in which two (or more) diseases and health conditions cluster; (3) the clustering of diseases results in adverse disease interaction, either biological or social or behavioural, increasing the health burden of affected populations” [35]. It generally develops in the context of social disadvantage and inequality, so it is more commonly experienced by marginalized groups, such as MSM and TGW [16]. In one of the earliest empirical studies in this literature, Stall et al. [36] attempted to extend the conceptual thinking about syndemic to understanding the HIV risk in MSM in the United States [37].

For a long time, TGW, a subgroup with different behavioral characteristics, have often been coded as MSM in behavioral and disease surveillance studies without targeted interventions [3]. The relationship between overlapping psychosocial health problems faced by TGW and HIV risk behaviors is unique to this population and requires specific interventions [38]. In fact, there is insufficient research guided by the syndemic framework to explore sexual risk behaviors among TGW. Several previous studies on the utility of syndemic theory have included stimulant use, heavy alcohol use, depression, poly-drug use, child sex abuse (CSA), IPV, and transgender-specific victimization in the framework, and these factors were positively correlated with each other and increased the odds of sexual risk behaviors [13,16,38,39]. In a recent study published in 2021, She et al. interviewed 204 transgender women sex workers (TGSW) in Shenyang, China [40]. This was the first study conducted among transgender women in China to explore the association between psychosocial problems and sexual risk behaviors, and it indicated that discrimination, victimization, rejection, anxiety, and life dissatisfaction are significantly related to CAS. In addition to these factors verified in most studies, further exploration was necessary to detect potential psychosocial problems that may play an important role in the syndemic effect on sexual risk behaviors. Therefore, low self-esteem, loneliness, and sexual compulsivity were added in our study since these psychosocial problems are highly prevalent in TGW and have been demonstrated to be associated with sexual risk behaviors.

Based on the syndemic framework and the evidence of previous research, we sought to contribute to addressing the literature gaps in potential psychosocial variables associated with sexual risk behaviors. In this study, we explored six psychosocial problems (low self-esteem, anxiety, depression, loneliness, sexual compulsivity, and IPV). This study aims to examine whether these factors co-occur and interact to increase the risk of engaging in CAS among TGW in China.

## 2. Methods

### 2.1. Participants and Study Setting

A cross-sectional study was conducted in Shenyang and Kunming, China, from November 2018 to January 2019. Shenyang is located in the north-eastern region of China, with a relatively high level of economic development and convenient transportation, and is an important gathering place for TGW to go to the south for work and to return home in the north. Kunming, a city with a relatively high degree of economic development in the southwest, has also attracted a large number of TGW due to its historical roots. Inclusion criteria included (1) at least 18 years old; (2) assigned the male sex at birth but presently identifying herself as a woman or transwoman; (3) had anal intercourse in the past 6 months; and (4) voluntary participation in the study and willingness to provide written informed consent.

### 2.2. Study Procedure

TGW in China often hide themselves to avoid being discriminated against by society, so it is difficult to use the probability sampling method to recruit eligible participants. Instead, we used snowball sampling, an efficient way to reach the minority population, to carry out our study. Initially, in collaboration with the non-governmental organization (NGO) dedicated to improving the physiological and psychological health of transgender women, we recruited five individuals as “seeds” to participate in our study. We then asked them to invite other eligible people to take part in the study. The procedure was repeated until participants could not invite others who met the inclusion criteria.

In total, 247 TGW were involved in our study. After providing informed consent, participants completed an anonymous questionnaire in a private room. During the process, a trained staff of NGO was there to offer help if participants had any trouble understanding the questions in the questionnaire. Each survey lasted about 30 min, and each participant received CNY 200 (approximately USD 30) for compensation after completing the survey. Moreover, individuals were invited to take a voluntary HIV rapid test provided by the local Centers for Disease Control and Prevention. In this study, all participants agreed to take the HIV test.

### 2.3. Ethical Considerations

The study was approved by the Ethics Committee of the School of Public Health, Shanghai Jiao Tong University, China. Before filling out the questionnaire participants were provided with informed consent forms, which described the study goals, procedures, and potential risks. If participants had any doubt, they were free to ask for help, as well as withdraw from the study at any time without being questioned. Access to the services of the institution was not affected by their refusal to participate.

### 2.4. Measures

#### 2.4.1. Sociodemographic Characteristics

Sociodemographic information included the participants’ age, education, monthly income, marital status, residential status, self-reported sexual orientation, and HIV-testing outcome. The test outcome was confirmed in the laboratory to reflect the real HIV status of the participants.

#### 2.4.2. Psychosocial Variables

Self-esteem. Self-esteem was measured using the 10-item Rosenberg’ Self-esteem Scale [41]. This scale is the most commonly used measure of self-esteem in psychiatric and psychological research to assess an individual’s self-worth. Total scores range from 0 to 30, and higher scores indicate higher self-esteem. Scores under 15 are considered a group of low self-esteem [42]. Cheung and Lau translated the scale into the Chinese version [43], and the scale was demonstrated to have good validity and reliability [44]. In this study, Cronbach’s α for this scale was 0.80.

Anxiety. Anxiety was assessed using the Generalized Anxiety Disorder (GAD-7), a 7-item, 4-point Likert scale. Total scores range from 0 to 21, with higher scores indicating higher levels of anxiety. A cut-off score of 10 was used to divide participants into groups with high and low levels of anxiety [45]. Cronbach’s α for this scale in this study was 0.92.

Depression. The Patient Questionaire-9 (PHQ-9), which comprises 9 items, was adopted to measure the depressive symptoms experienced by TGW in the past 2 weeks. The scores range from 0 to 27. Higher scores indicate more severe depressive symptoms, and 10 was recommended as a cut-off for “clinically significant” symptoms [46]. Previous studies have proven good validity and reliability of the Chinese version in the general population [47]. Additionally, in this study, Cronbach’s α for this scale was 0.92.

Loneliness. Loneliness was accessed using the 8-item UCLA Loneliness Scale (ULS-8) [48]. Responses are scored on a 4-point Likert scale ranging from 1 to 4, with total scores ranging from 8 to 32. As there is no existing recommendation for the cut-off for this scale, we used ascore of 19 (75th percentile) to identify those who have a high level of loneliness. Cronbach’s α for this scale in this study was 0.86.

Sexual compulsivity. Sexual compulsivity was measured using the 10-item Sexual Compulsivity Scale [49]. The scale uses a 4-point Likert response, with the sum of scores ranging from 10 to 40. Higher scores suggest higher levels of sexual compulsivity; a score of 24 and above indicates significant sexual compulsivity [50]. In this study, Cronbach’s α for this scale was 0.92.

Intimate partner violence. Four questions were used to measure whether TGW experienced violence victimization during their lifetime [27]: (1) physical violence: “Has your partner physically assaulted you (e.g., hit, pat, slap, push, kick, etc.)?”; (2) emotional violence: “Has your partner asked you to do anything inconsistent with your gender identity (such as not letting you use your makeup)?”; (3) verbal violence: “Has your partner ever verbally harassed, slandered, or threatened you?”; and (4) sexual violence: “Has your partner ever forced you to have sex?” Responses were rated on a 5-point Likert scale (1 = never to 5 = always). If participants reported “never” for each item, we defined them as not experiencing IPV. A dichotomous variable was created on the basis of these 4 items, with 0 suggesting no intimate partner violence and 1 suggesting any type of intimate partner violence. This measurement of violence victimization has been validated and widely used in violence studies [27,51].

#### 2.4.3. Condomless anal Sex

Participants were asked about the frequency of condom use with any type of sexual partner in both receptive and insertive anal sex during the previous 6 months. Responses ranged from 1 (never) to 4 (every time). Participants who responded with “4 (every time)” in the context of each type of sexual partner were defined as not engaging in CAS, while others were coded as participating in CAS.

### 2.5. Statistical Analysis

Statistical analysis was performed using SPSS version 26.0 and R version 4.1.1. We analyzed data in five steps. First, descriptive statistics was used to summarize the sociodemographic characteristics, prevalence of psychosocial problems, and sexual risk behaviors among transgender women. Second, univariate analysis was implemented by binary logistic regression to explore the association between sociodemographic characteristics and CAS. Third, we performed logistic regression analysis of association among six psychosocial variables to assess whether they clustered together. Fourth, we examined the association between psychosocial problems and CAS by both univariate and multivariate logistic regression models. Lastly, we used multivariable logistic regression models to test pairwise interactions on an additive scale of the syndemic factors that were statistically significant in step 4 as predictors of CAS. Additive interaction, also known as synergism, refers to the interactive effect of two or more risk factors and is considered an appropriate way of accounting for the underlying causal mechanisms of a given outcome in health behavior research and therefore has more direct public health relevance [37,52]. We did not test greater than two-way interaction due to the consideration of small counts within levels of the syndemic factors. Three indices developed by Rothman [53] were computed to test the interaction contrast using the epiR package in R. The relative excess risk due to interaction (RERI) was used to compare the interactive effect of psychosocial problems on an additive scale [54]. The attributable proportion due to interaction (AP) computes the attributable proportion of risk due to interaction among those with both psychosocial problems. The synergy index (S) was used to determine the direction of the interactive effect. Some epidemiologists consider any departure from 0 (in the case of RERI and AP) or 1 (in the case of S) as evidence for the presence of interaction [52]. To measure synergism metrics, three dummy variables were created to calculate the joint effects of each pair of the syndemic factors [55,56]. The dummy variables were coded as follows: 0 = presence of neither factor (reference category); 1 = presence of factor A but not factor B; 2 = presence of factor B but not condition A; and 3 = presence of both factors.

## 3. Results

### 3.1. Sociodemographic and Psychosocial Characteristics of Participants

The sociodemographic and psychosocial characteristics of 247 TGW who agreed to participate and completed the whole questionnaire are presented in Table 1. The participants had an average age of 33.0 (±8.1) years. Most participants (68.8%) had an education of senior high school or less, and 29.1% earned less than CNY 3000 per month. In addition, 19.4% of them reported being HIV positive, and 11.7% of the participants were characterized as having low self-esteem, while 10.5% reported having generalized anxiety disorder. Of all participants, 23.5% were found to have depression and 23.9% reported that they felt lonely. High sexual compulsivity was reported by 32.8% of the participants. In addition, the prevalence of intimate partner violence was 64.8%. Furthermore, 30.8% of the participants reported that they had condomless anal sex in the past 6 months.

### 3.2. Association between Sociodemographic Characteristics and CAS

The result of univariate logistic regression between sociodemographic characteristics and CAS is summarized in Table 2. Participants who had a college degree or above were less likely to report CAS compared to those who had a poorer educational background (OR = 0.53, 95% CI = 0.29–1.00). Those with a monthly income of more than CNY 6000 were less likely to participate in CAS than those who earned less than CNY 3000 (OR = 0.35, 95% CI = 0.17–0.74). Those who reported being divorced or widowed were more likely to engage in CAS when compared to single individuals (OR = 3.35, 95% CI = 1.51–7.42). Additionally, HIV-positive participants were more likely to engage in CAS (OR = 3.94, 95% CI = 2.04–7.58).

### 3.3. Co-Occurrence among Psychosocial Problems

As illustrated in Table 3, psychosocial problems showed a considerable degree of clustering. Participants who reported low self-esteem were more likely to report depression (AOR = 7.36, 95% CI = 3.08–17.60) and loneliness (AOR = 7.04, 95% CI = 3.05–16.27). Participants who reported anxiety were more likely to report depression (AOR = 15.08, 95% CI = 5.65–40.28), loneliness (AOR = 13.91, 95% CI = 5.25–36.90), sexual compulsivity (AOR = 9.58, 95% CI = 3.58–25.60), and experiencing IPV (AOR = 2.44, 95% CI = 1.06–5.62). Depression was significantly associated with loneliness (AOR = 7.62, 95% CI = 3.80–15.27), sexual compulsivity (AOR = 3.69, 95% CI = 1.96–6.96), and experiencing IPV (AOR = 3.33, 95% CI = 1.79–6.22). Participants who reported loneliness also reported sexual compulsivity (AOR = 2.65, 95% CI = 1.42–4.93) and IPV (AOR = 2.16, 95% CI = 1.18–3.95). Sexual compulsivity was significantly associated with IPV (AOR = 3.26, 95% CI = 1.84–5.77).

### 3.4. Association between Psychosocial Variables and CAS

The relationship between psychosocial problems and CAS after adjusting for education, monthly income, marital status, and HIV-testing outcome is shown in Table 4. Our analysis displayed that four psychosocial variables were statistically significant. A lower level of self-esteem (AOR = 3.14, 95% CI = 1.34–7.34), a higher level of depression (AOR = 2.53, 95% CI = 1.29–4.96), a higher level of sexual compulsivity (AOR = 2.71, 95% CI = 1.47–5.00), and being subjected to IPV (AOR = 2.81, 95% CI = 1.54–5.12) indicated an increasing probability of engaging in CAS. As shown in the results from multivariate logistic regression, three psychosocial variables still remained significant: low self-esteem (ORm = 2.99, 95% CI = 1.25–7.18), sexual compulsivity (ORm = 2.13, 95% CI = 1.13–4.00), and IPV (ORm = 2.21, 95% CI = 1.19–4.11).

### 3.5. Pairwise Interaction of Psychosocial Variables on CAS

The interactive effects of psychosocial problems are shown in Table 5. The joint effect of low self-esteem and sexual compulsivity was significantly and positively associated with higher odds of CAS compared to those with neither factor (AOR = 4.42, 95% CI = 1.15–17.56). Similarly, the joint impact of low self-esteem and IPV (AOR = 5.48, 95% CI = 1.72–18.55) as well as sexual compulsivity and IPV (AOR = 4.95, 95% CI = 2.24–11.19) was related to increasing the odds of CAS in comparison to those who reported neither factor respectively. Surprisingly, our test indicated no interactive effects of three pairwise psychosocial factors, as all the RERI as well as AP were not significantly different from 0 and S were not different from 1.

## 4. Discussion

The prevalence of HIV among TGW in our study was 19.4%, which is close to the worldwide prevalence of HIV (19.1%) [2] and that reported by another study conducted in Shenyang, China (18.1%) [40]. Condomless anal sex is the highest-risk behavior for HIV transmission and acquisition because the rectal mucosa is highly vulnerable and lacks local humoral immune protection [27]. In this study, 30.8% TGW reported that they had experienced CAS in the past 6 months, which is lower than that reported in previous studies in China [3,57]. Our findings verified that TGW are at high risk of HIV infection, so it is urgent to provide effective HIV preventive interventions to them for reducing high-risk sexual behaviors and alleviating the disease burden within the population.

We found that four sociodemographic factors may be associated with CAS among TGW, among which education, divorce, and HIV-positive status were consistent with another study in China [40]. Higher education was detected to be a protective factor against CAS. This may be explained by the fact that people with higher education are more likely to have knowledge about how to take care of themselves. Those earning more had lower odds of engaging in CAS, possibly because people with good financial ability tend to have better access to health information and resources, which makes them more aware of the harm of sexual risk behaviors. According to the reserve capacity model, limited cognitive coping resources restrict individuals experiencing financial hardship and socioeconomic disadvantage from coping with excessive stressors, leading to participation in maladaptive coping behaviors, such as engaging in condomless sexual behaviors [58]. Our results indicated that divorce or death of a spouse is associated with higher odds of sexual risk taking. It is well documented that those who are divorced or widowed experience higher levels of mental health problems [59,60], which induces them to resort to sexual risk behaviors as a way of dealing with emotional distress. HIV seropositivity was significantly related to CAS in this study, which verified the consensus that there is a strong connection between HIV infection and sexual risk behaviors.

The first principle in syndemic theory is that two or more health conditions co-occur within a population. Consistent with the majority of studies [61,62], psychosocial problems are shown to be clustered together. Four psychosocial factors were discovered to be associated with CAS among TGW in our study, indicating that close attention should be paid to the relationship between psychosocial health problems and sexual risk behaviors. Low self-esteem, sexual compulsivity, and IPV were statistically significant in both univariate and multivariate logistic regression models, while depression was detected to be associated with CAS in the univariate logistic regression model. We found that lower levels of self-esteem are linked to participating in CAS. TGW with low self-esteem may not feel empowered to use condoms consistently, and qualitative research proved that some TGW regard engaging in sex work and unprotected sex as a way of building self-esteem [21,63]. Depression is associated with low self-esteem, and individuals may engage in sexual risk behavior to satisfy their sexual and narcissistic needs to obtain a sense of control and to compensate for low levels of self-esteem [64]. Sexual compulsivity is characterized as an inclination to engage in sexually related activities, with a higher level indicating one’s preoccupation with sex and an inability to control sexual impulses [65]. There is much evidence of the relationship between sexual compulsivity and sexual risk behaviors in MSM [32,65,66], yet little research has focused on transgender populations. Although the specific causal relationship is unknown, our study has extended the literature that sexual compulsivity is a strong predictor for engaging in CAS among TGW. We also provided further evidence that experiencing IPV is a risk factor of participating in CAS, which is in accordance with previous studies both on MSM [67] and on TGW [28]. Newcomb et al. [68] considered that the association between IPV and CAS is actuated by the power imbalance. Specifically, individuals who have experienced IPV find it more difficult to negotiate safe sexual practices with their partners due to their feelings of insecurity [67].

In a few prior studies conducted among MSM [33,61], the interactive effects of psychosocial problems on CAS or HIV infection were observed. However, limited empirical studies were carried out among transgender populations. A previous study conducted among TGSW in China reported non-significant findings [40]. Similarly, we found no evidence of a significant interactive effect of pairwise psychosocial factors on sexual risk behaviors, which may be partly attributed to the exclusion of other syndemic factors and the small sample size in our study. Future investigations are required to determine whether co-occurring psychosocial conditions would lead to an expansion of sexual risks among transgender populations [40].

In addition to exploring the potential psychosocial problems existing among TGW, future research needs to examine how these health conditions work synergistically—the central principle to syndemic theory to increase sexual risk behaviors through large samples. Only by understanding how syndemic factors play out can effective preventive interventions be developed. In the past, HIV prevention interventions always lay emphasis on merely changing sexual risk behaviors, ignoring the important role psychosocial problems play in affecting sexual risk behaviors. Considering the interactive effects of psychosocial problems, it may be more cost-effective to target specific conditions where expansion is most risky in low-resource settings with evidence of synergy, while multi-component interventions that focus on all or most of the individual syndemic conditions are more effective in the absence of synergy [33]. Only a few HIV risk reduction interventions are designed specifically for TGW. TransAction, which originated in Los Angeles County, is a locally developed multi-component intervention that uses individual- and group-level interventions for TGW and has proved to be effective in reducing HIV sexual risk behaviors by integrating responses to the syndemic of co-occurring health disparities [69]. Additively, implementing effective interventions to reduce sexual risk behaviors among marginalized populations requires not only programmatic improvement but also attention to broader social and policy contexts that can alleviate adverse health conditions [52].

## 5. Limitations

Although our study makes a contribution to the understanding of the syndemic effects of psychosocial problems related to CAS among TGW in China, there are several limitations. First, as our study was a cross-sectional study, it was hard to verify the cause-and-effect relationship between psychosocial health problems and engaging in CAS, which suggests that prospective studies should be conducted in the future to figure out the causality. Second, selection bias was inevitable as the result of applying snowball sampling during the recruitment of participants. Our research only included those who were willing to participate in the investigation and excluded those who refused to take part. Third, considering the TGW involved in our study came from two cities in China, our conclusions may not apply to other countries due to culture differences. Fourth, the subjectivity of feedback from interviewers may cause recall and self-report bias. Fifth, although we incorporated psychosocial variables as much as possible, other potential syndemic factors may not have been included in our study. Finally, due to the relatively small sample size, we only considered the behavior of CAS but ignored the different types of partners involved in CAS, so future studies with larger populations are required to explore CAS with different partners.

## 6. Conclusions

Our study extended the literature on the prevalence of specific psychosocial problems and sexual risk behaviors among TGW in China. Importantly, evidence that psychosocial problems have a great impact on sexual risk behaviors was observed in this study. These findings imply that programmatic and effective HIV prevention interventions addressing psychosocial problems are required for TGW.

## Figures and Tables

**Table 1:** Sociodemographic characteristics of transgender women (*N* = 247).

Characteristics	*N*	%
Age	33.0 ± 8.1	
<30	87	35.2
30–45	137	55.5
>45	23	9.3
Education		
Senior high school or less	170	68.8
College degree or above	77	31.2
Monthly income (CNY ^a^)		
<3000	72	29.1
3000–6000	105	42.5
>6000	70	28.3
Marital status		
Single	201	81.4
Married	17	6.9
Divorced or widowed	29	11.7
Residential status		
Local	93	37.7
Nonlocal	154	62.3
Self-reported sexual orientation		
Heterosexual	37	15
Homosexual	139	56.3
Bisexual	44	17.8
Pansexual	13	5.3
Unsure	14	5.7
HIV positive		
Yes	48	19.4
No	199	80.6
Rosenberg Self-Esteem Scale		
High level	218	88.3
Low level	29	11.7
GAD (anxiety)		
Low level	221	89.5
High level	26	10.5
PHQ-9 (depression)		
Low level	189	76.5
High level	58	23.5
UCLA Loneliness Scale		
Low level	188	76.1
High level	59	23.9
Sexual compulsivity		
Low level	166	67.2
High level	81	32.8
IPV		
Yes	160	64.8
No	87	35.2
CAS		
Yes	171	31.8
No	76	68.2

^a^ CNY 3000 equivalent to USD 466; CNY 6000 equivalent to USD 931. IPV: intimate partner violence; CAS: condomless anal sex.

**Table 2:** Association between sociodemographic characteristics and CAS among transgender women (*N* = 247).

Characteristics	*N*	%	ORu (95% CI)
Age			
<30	20	23.0	Ref
30–45	47	34.3	1.75 (0.95–3.22)
>45	9	39.1	2.15 (0.81–5.71)
Education			
Senior high school or less	59	34.7	Ref
College degree or above	17	22.1	0.53 (0.29–1.00) *
Monthly income (CNY ^a^)			
<3000	30	41.7	Ref
3000–6000	32	30.5	0.61 (0.33–1.15)
>6000	14	20.0	0.35 (0.17–0.74) **
Marital status			
Single	54	26.9	Ref
Married	6	35.3	1.49 (0.52–4.21)
Divorced or widowed	16	55.2	3.35 (1.51–7.42) **
Residential status			
Local	25	26.9	Ref
Nonlocal	51	33.1	1.35 (0.76–2.38)
Self-reported sexual orientation			
Heterosexual	10	27.0	Ref
Homosexual	46	33.1	1.34 (0.60–2.99)
Bisexual	12	27.3	1.01 (0.38–2.71)
Pansexual	4	30.8	1.20 (0.30–4.79)
Unsure	4	28.6	1.08 (0.28–4.24)
HIV positive			
Yes	49	24.6	Ref
No	27	56.3	3.94 (2.04–7.58) **

ORu: univariate odds ratio. ^a^ CNY 3000 equivalent to USD 466; CNY 6000 equivalent to USD 931. * *p* < 0.05, ** *p* < 0.01.

**Table 3:** Adjusted associations of psychosocial problems among transgender women (*N* = 247).

	AOR (95% CI)					
	Low Self-Esteem	Anxiety	Depression	Loneliness	Sexual Compulsivity	IPV
Low Self-Esteem	——					
Anxiety	2.74 (0.97–7.78)	——				
Depression	7.36 (3.08–17.60) ***	15.08 (5.65–40.28) ***	——			
Loneliness	7.03 (3.05–16.27) ***	13.91 (5.25–36.90) ***	7.62 (3.80–15.27) ***	——		
Sexual Compulsivity	1.26 (0.55–2.88)	9.58 (3.58–25.60) ***	3.69 (1.96–6.96) ***	2.65(1.42–4.93) **	——	
IPV	2.13 (0.96–4.69)	2.44 (1.06–5.62) *	3.33 (1.79–6.22) ***	2.16 (1.18–3.95) *	3.26 (1.84–5.77) ***	——

AOR: adjusted odds ratio, adjusted for education, monthly income, marital status, and HIV-testing outcome; IPV: intimate partner violence. * *p* < 0.05, ** *p* < 0.01, *** *p* < 0.001.

**Table 4:** Association between psychosocial variables and CAS among transgender women (*N* = 247).

Variables	CAS				
	*N*	%	ORu (95% CI)	AOR (95% CI)	ORm (95% CI)
Rosenberg Self-Esteem Scale					
High level	60	27.5	Ref	Ref	Ref
Low level	16	55.2	3.24 (1.47–7.14)	3.14 (1.34–7.34) **	2.99 (1.25–7.18) *
GAD (anxiety)					
Low level	67	30.3	Ref	Ref	
High level	9	34.6	1.22 (0.52–2.87)	1.43 (0.58–3.56)	
PHQ-9 (depression)					
Low level	49	25.9	Ref	Ref	
High level	27	46.6	2.49 (1.35–4.58)	2.53 (1.29–4.96) **	
UCLA Loneliness Scale					
Low level	53	28.2	Ref	Ref	
High level	23	39	1.63 (0.88–3.00)	1.71 (0.89–3.29)	
Sexual compulsivity					
Low level	39	23.5	Ref	Ref	Ref
High level	37	45.7	2.74 (1.56–4.82)	2.71 (1.47–5.00) **	2.13 (1.13–4.00) *
IPV					
Yes	37	23.1	Ref	Ref	Ref
No	39	44.8	2.70 (1.54–4.73) **	2.81 (1.54–5.12) **	2.21 (1.19–4.11) *

ORu: univariate odds ratio; AOR: adjusted odds ratio, adjusted for education, monthly income, marital status, and HIV-testing outcome; ORm: multivariate odds ratio; CAS: condomless anal sex. * *p* < 0.05, ** *p* < 0.01.

**Table 5:** Pairwise interaction of psychosocial variables on CAS among transgender women (*N* = 247).

		AOR (95% CI)	RERI (95% CI)	AP (95% CI)	S (95% CI)
Low Self-Esteem	Sexual Compulsivity				
-	-	Ref	−2.47 (−10.29–5.36)	−0.50 (−2.55–1.55)	0.61 (0.11–3.31)
+	-	4.97 (1.67–15.11) **			
-	+	3.15 (1.62–6.18) ***			
+	+	4.42 (1.15–17.56) *			
Low Self-Esteem	IPV				
-	-	Ref	0.17 (−7.18–7.53)	−0.03 (−1.21–1.27)	0.93 (0.22–4.90)
+	-	3.90 (1.11–13.42) **			
-	+	2.90 (1.51–5.66) **			
+	+	5.48 (1.72–18.55) **			
Sexual Compulsivity	IPV				
-	-	Ref	0.17 (−7.18–7.53)	−0.03 (−1.21–1.27)	0.93 (0.22–4.90)
+	-	3.90 (1.11–13.42) **			
-	+	2.90 (1.51–5.66) **			
+	+	5.48 (1.72–18.55) **			

AOR: adjusted odds ratio, adjusted for education, monthly income, marital status, and HIV-testing outcome. * *p* < 0.05, ** *p* < 0.01, *** *p* < 0.001; -: absence of factor; +: presence of factor.

## Data Availability

The data presented in this study are available on reasonable request from the corresponding author.
